# 1771. A Novel Approach to Distributing COVID-19 Oral Antivirals During a Public Health Emergency Using a Pharmacy Home Delivery Model in New York City, 2022

**DOI:** 10.1093/ofid/ofad500.1601

**Published:** 2023-11-27

**Authors:** Mary M K Foote, Elizabeth Diago Navarro, Matthew Silverstein, Niroshan Shanmugarajah, Meredith Eddy, Ramona Lall, Pierre J Amiel, Iris Cheng, Joseph Kennedy, Hiu Tai Chan

**Affiliations:** New York City Department of Health and Mental Hygiene, LONG ISLAND CITY, New York; New York City Department of Health and Mental Hygiene, LONG ISLAND CITY, New York; New York City Department of Health and Mental Hygiene, LONG ISLAND CITY, New York; New York City Department of Health and Mental Hygiene, LONG ISLAND CITY, New York; New York City Department of Health and Mental Hygiene, LONG ISLAND CITY, New York; New York City Department of Health and Mental Hygiene, LONG ISLAND CITY, New York; New York City Department of Health and Mental Hygiene, LONG ISLAND CITY, New York; New York City Department of Health and Mental Hygiene, LONG ISLAND CITY, New York; NYC Department of Health, New York, New York; NYC Department of Health, New York, New York

## Abstract

**Background:**

In December 2021, the oral antivirals (OAVs) nirmatrelvir/ritonavir and molnupiravir were authorized for the treatment of COVID-19 in high-risk patients. Initial supplies were limited and states designated pharmacies to receive OAVs from a weekly federal allocation. New York City (NYC) has nearly 3,000 pharmacies serving a diverse population across a large urban area. To ensure fair access to OAVs, the NYC Health Department partnered with an online pharmacy to provide rapid delivery to any NYC address. Here we describe characteristics of patients served and metrics used to evaluate the program as the pandemic and OAV access evolved in 2022.

**Methods:**

The NYC partner pharmacy provided data on OAV prescriptions including patient age, sex, delivery zip code, and dates of prescription and delivery. These data were matched with the NYC Health Department’s COVID-19 surveillance data including positive lab reported cases, Citywide Immunization Registry, deaths, all-cause emergency department (ED) visits and hospital admissions. Descriptive statistics were calculated using R Statistical Software.

**Results:**

A total of 48,947 OAV prescriptions were delivered in 2022, reaching 99.5% of residential zip codes, accounting for 17.5% of the OAVs dispensed in NYC. The median time from prescription receipt to delivery was 17 hours (IQR 6-24) with half (49%) of deliveries going to high social vulnerability zip codes. Of OAV recipients, most (58%) were female, with a median age of 56 years (IQR 39, 68) with 32% aged 65 years or older; 78% had received at least a primary vaccination series; 36% matched with a lab reported COVID-19 case; 5.5% had an ED visit and 0.8% were hospitalized in NYC for any cause within 30 days of prescription date.

**Figure:**
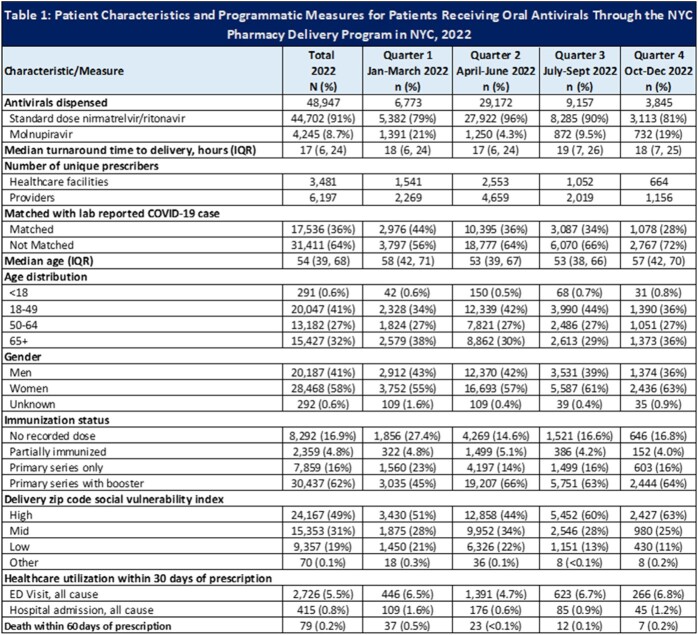

**Conclusion:**

The NYC antiviral delivery program provided broad and timely access to COVID OAVs, serving a significant proportion of patients in high social vulnerability neighborhoods and older adults at higher risk for severe outcomes. Of note, an increasing proportion of people receiving OAVs were not reported as cases to public health, which likely reflects the increasing use of home tests in 2022. Limitations include the lack of data on race/ethnicity, underlying medical conditions and for patients receiving OAVs through other pharmacies.

**Disclosures:**

**All Authors**: No reported disclosures

